# Reduction of inflammation and mitochondrial degeneration in mutant SOD1 mice through inhibition of voltage-gated potassium channel Kv1.3

**DOI:** 10.3389/fnmol.2023.1333745

**Published:** 2024-01-16

**Authors:** Patrizia Ratano, Germana Cocozza, Cecilia Pinchera, Ludovica Maria Busdraghi, Iva Cantando, Katiuscia Martinello, Mariarosaria Scioli, Maria Rosito, Paola Bezzi, Sergio Fucile, Heike Wulff, Cristina Limatola, Giuseppina D’Alessandro

**Affiliations:** ^1^IRCCS Neuromed, Pozzilli, Italy; ^2^Department of Physiology and Pharmacology, University of Rome Sapienza, Rome, Italy; ^3^Department of Fundamental Neurosciences, University of Lausanne, Lausanne, Switzerland; ^4^Department of Pharmacology, University of California Davis, Health Sciences Drive, Davis, CA, United States; ^5^Department of Physiology and Pharmacology, Laboratory Affiliated to Istituto Pasteur, Sapienza University, Rome, Italy

**Keywords:** ALS, mutant SOD1, Kv1.3 channels, mitochondria, inflammation

## Abstract

Amyotrophic lateral sclerosis (ALS) is a fatal neurodegenerative disease with no effective therapy, causing progressive loss of motor neurons in the spinal cord, brainstem, and motor cortex. Regardless of its genetic or sporadic origin, there is currently no cure for ALS or therapy that can reverse or control its progression. In the present study, taking advantage of a human superoxide dismutase-1 mutant (hSOD1-G93A) mouse that recapitulates key pathological features of human ALS, we investigated the possible role of voltage-gated potassium channel Kv1.3 in disease progression. We found that chronic administration of the brain-penetrant Kv1.3 inhibitor, PAP-1 (40 mg/Kg), in early symptomatic mice (i) improves motor deficits and prolongs survival of diseased mice (ii) reduces astrocyte reactivity, microglial Kv1.3 expression, and serum pro-inflammatory soluble factors (iii) improves structural mitochondrial deficits in motor neuron mitochondria (iv) restores mitochondrial respiratory dysfunction. Taken together, these findings underscore the potential significance of Kv1.3 activity as a contributing factor to the metabolic disturbances observed in ALS. Consequently, targeting Kv1.3 presents a promising avenue for modulating disease progression, shedding new light on potential therapeutic strategies for ALS.

## 1 Introduction

Amyotrophic lateral sclerosis (ALS) is a fatal neurodegenerative disease, that causes progressive loss of motor neurons in the spinal cord, brainstem, and motor cortex. Most cases of ALS are sporadic (SALS); about 10% are familial (FALS), some of them carrying mutant forms of Cu, Zn superoxide dismutase 1 (SOD1) (FALS1), recognized to promote a gain of neurotoxic function (Poppe et al., 2014). In 90% of sporadic cases, one of the challenging aspects of human ALS is that the diagnosis is only made when the pathology is already symptomatic. However, ALS starts long before symptom onset (Mitsumoto et al., 2014) and there is an urgent need to hasten the diagnosis as well as to search for therapeutic strategies effective at the symptomatic stage of the disease.

Kv1.3 is a voltage-gated K+ channel originally described in human T cells and suggested as a target for immunosuppression (DeCoursey et al., 1984). Kv1.3 activity allows for the K+ efflux that is necessary to sustain Ca2+ entry through Ca2+ release-activated channels and to initiate downstream signaling and cytokine production (Negulescu et al., 1994). The Kv1.3 channels are almost ubiquitously expressed on the plasma membrane of T and B lymphocytes, macrophages, fibroblasts, platelets, osteoclasts, microglia, oligodendrocytes, and on different organs and tissues, among them brain, lung and, pancreas. In specific cell subsets, such as T lymphocytes, cancer cells, and more recently in auditory and medium spiny neurons the channels are also expressed in the inner mitochondrial membrane (Szabò et al., 2005; Bednarczyk, 2009; Bielanska et al., 2009; Gazula et al., 2010; Otuyemi et al., 2023). In T lymphocytes, mitochondrial Kv1.3 plays an important role in the sequence of events leading to Bax-induced cytochrome c release (Gulbins et al., 2010).

Recently, the activation of Kv1.3 channels has been implicated in many neurodegenerative diseases, including Alzheimer’s disease, Parkinson’s disease, and multiple sclerosis, where its inhibition has been reported to be beneficial. In these scenarios, it has been shown that the activation of the channels on microglia correlates with an inflammatory phenotype, with a detrimental effect on neurons (Rangaraju et al., 2009; Chen et al., 2017; Maezawa et al., 2018; Sarkar et al., 2020; Cojocaru et al., 2021). Whether Kv1.3 also contributes to the pathogenesis of ALS has not been studied, nor has the effect of inhibiting it in animal models of the disease. Glial cells play important roles in the death of motor neurons, and the multicellular aspects of ALS disease are supported by several evidence (Eisen, 1995; Hall et al., 1998; Boillée et al., 2006; Dobrowolny et al., 2008; Yamanaka et al., 2008a,b; Lobsiger et al., 2009).

Both central and peripheral inflammation have been implicated in the onset and progression of ALS, and signs of inflammation are reported in animal models and patient tissues (Hall et al., 1998; Turner et al., 2004; Mantovani et al., 2009; Sweeney et al., 2019; Wu et al., 2020; Pan and Nicolazzo, 2022). Additional mechanisms, including mitochondrial dysfunction, have been involved in the pathogenesis of ALS (Mejzini et al., 2019). Fragmentation of mitochondria, dysfunctions, and morphological alterations are described (Sasaki et al., 2007) as well as decreased respiratory activity in freshly isolated mitochondria from the spinal cords of hSOD1-G93A mice (Jung et al., 2002; Mattiazzi et al., 2002). Mutant SOD1 aggregates at the outer membrane of mitochondria, inactivating the anti-apoptotic protein Bcl-2 (Pedrini et al., 2010) resulting in the release of cytochrome c possibly triggering mitochondrial intrinsic apoptosis (Pasinelli et al., 2000, 2004).

In the present study, we investigated the hypothesis that the activity of Kv1.3 channels could modulate mitochondrial function in the progression of ALS. For this study, we used the human mutant superoxide dismutase-1 (hSOD1-G93A) mouse which resembles several features of the human pathology and is a widely used model to investigate the mechanisms and to test new possible therapies (Julien and Kriz, 2006).

To verify our hypothesis, we treated hSOD1G93A mice, at the symptomatic stage, with a brain-penetrant channel inhibitor, 5-(4-phenoxybutoxy)psoralen (PAP-1), and observed reduced motor deficits, reduction of astrocyte reactivity and microglial Kv1.3 expression in the lumbar ventral horns of the spinal cord. We also observed that Kv1.3 inhibition significantly extends the survival and reduces pro-inflammatory factors in the peripheral blood of hSOD1-G93A mice. In addition, we described the expression of Kv1.3 in lumbar spinal cord mitochondria and reported that channel inhibition ameliorated the structural mitochondria deficits and increased the oxygen consumption rate in cultured motor neurons. Taken together, these data suggest that the activity of the Kv1.3 in mitochondria could represent one of the mechanisms involved in the metabolic impairment reported in ALS.

## 2 Materials and methods

### 2.1 Animal model

All procedures used in this study were approved by the Italian Ministry of Health (Approval No. 374/2018-PR) according to the ethical guidelines for animal use in EC Council Directive 2010/63/EU and Italian D.Leg 26/2014. Male B6SJL-Tg(SOD1-G93A)1Gur/J (SOD1-G93A) and B6SJL-Tg(SOD1)2Gur/J (control) mice (Jackson Laboratory, Bar Harbor, ME, USA; stock n. 002726) served as subjects in this experiment. Only male mice were used for the experiments to minimize gender-induced differences in motor impairment and survival (Choi et al., 2008). B6SJL-Tg(SOD1)2Gur/J mice carry a high copy number of the mutated allele of the human SOD1 gene. In contrast to low-expressing SOD1-G93A mice (also referred to as G1L), which exhibit delayed disease onset and mortality, the high-expressing SOD1-G93A mice (also referred to as G1H) used here typically survive only approximately 120 days. B6SJL-Tg(SOD1)2Gur/J were maintained by breeding male hemizygous carriers to B6SJLF1 female hybrids. B6SJLF1 female hybrids are the offspring of a cross between C57BL/6J females (B6) and SJL/J males (SJL) from Charles River Laboratories. DNA obtained from tail biopsies was used to identify transgenic mice by PCR. Briefly, tail tips were digested (overnight, 58°C) in a buffer containing 100 mM Tris-HCl pH 8, 0.1% SDS 20, 5 mM EDTA pH 8, 200 mM NaCl, and 20 mg/ml Proteinase K (Ambion-Thermo Fisher, Germany, #2548). Genomic DNA was amplified with SsoFast Eva Green Supermix (Bio-Rad, California, #172-5201) using the following primers: SOD1 forward 5′-CATCAGCCCTAATCCATCTGA-3′; SOD1 reverse 5′-CGCGACTAACAATCAAAGTGA-3′. Mice were group-housed (two or three per cage) in regular polycarbonate cages (30 × 16 × 11 cm), at constant temperature (22 ± 1°C) and humidity (50%). Mice were maintained on a 12/12 h light/dark cycle (light 7 a.m. to 7 p.m.) with *ad libitum* access to food and water. Body weight was recorded once a week beginning at 7 weeks of age. Starting at 7 weeks of age mice were evaluated for motor deficits.

### 2.2 PAP-1 treatment and survival analysis

PAP-1 (5-(4-phenoxybutoxy)psoralen) was synthesized as previously described (Schmitz et al., 2005). Mice were randomly assigned to the vehicle (50 μl, Mygliol-812, IOI Oleo GmbH, Hamburg, Germany) or PAP-1 (40 mg/Kg) treatment. The chosen treatment dose has shown efficacy in mouse models of Parkinson’s disease (Sarkar et al., 2020), Alzheimer’s disease (Maezawa et al., 2018) and stroke (Chen et al., 2017). In addition, in ischemic stroke in mice 40 mg/kg of PAP-1 was more effective than 10 mg/kg. Mice were treated daily (5 days/week) with intraperitoneal injections starting between 9 and 11 weeks of age. Animals were treated until the age described in the text or once the humane endpoint was reached for the survival analysis experiments. Animals were sacrificed when unable to stand up within 20 seconds after being placed on either side.

### 2.3 Behavioral assessment

Performance on a rotating rod, strength, and grip force was evaluated once a week usually between 9 a.m. to 3 p.m.

#### 2.3.1 Rotarod test

A rotarod apparatus (Ugo Basile, Gemonio Italy, #47650) was used to assess motor coordination, strength, and balance. Animals were placed on the rotating cylinder at a constant speed of 15 rpm. The arbitrary time limit was 300 seconds and the longest latency was recorded.

#### 2.3.2 Hanging wire test

Mice were tested for strength as previously described (Rinaldi et al., 2013).

#### 2.3.3 Grip strength test

The equipment consisted of a grip force measuring device (Ugo Basile, No. 47200), completed with a force transducer and a gripper (grid to measure the 4 limbs). The mouse was held at the base of the tail and allowed to grasp the grid with four limbs. The mouse was then gently moved backward until it released its grip. The peak force of each trial was taken as a measure of grip strength and values were normalized to mouse weight.

### 2.4 Immunofluorescence and Motor Neuron (MN) survival evaluation

At the end of the experiments, mice were overdosed with ketamine 300–360 mg/kg + xylazine 30-40 mg/kg i.p. and then intra-cardially perfused with PBS and then PFA 4%; spinal cord tissues were then isolated, fixed in 4% formaldehyde, cryopreserved in 30% sucrose solution and snap frozen. Cord sections (20μm) were rinsed in PBS, blocked (3% goat serum in 0.3% Triton X-100) for 1 h at RT, and incubated overnight at 4°C with specific antibodies diluted in PBS containing 1% goat serum and 0.1% Triton X-100. Sections were incubated with Hoechst for nuclear visualization and the following primary antibodies: Iba1 (Wako, Osaka Japan, #019-19741, 1:500), GFAP (Novus Biologicals, Littleton USA, #NB300-141, 1:500), SMI-32 (BioLegend, 1:500), CD8 (BD Pharmigen #553026, 1:50) and Kv1.3 (Alomone labs, Jerusalem Israel, #AGP-005, 1:200). After three washes, sections were incubated with secondary goat anti-rabbit AlexaFluor 594, anti-rabbit AlexaFluor 488, donkey anti-mouse AlexaFluor 488, anti-rat AlexaFluor 488, and goat anti-guinea-pig 594 (1:1,000 dilution, Thermo Fisher A-11037, A-11034, A-21202, A-11006, and A-11076, respectively) for 1 h at room temperature. Sections were washed, mounted, and analyzed by fluorescence microscopy.

For Iba1/Kv1.3/SMI-32 staining, coronal sections were first boiled for 20 min in citrate buffer (pH 6.0) at 95–100°C. Antibody reactivity was quantified in lumbar spinal cord sections (12 serial coronal sections for each animal, in each group, covering L3 to L5) using MetaMorph 7.6.5.0 image analysis software (Molecular Device, San Jose, USA) after background subtraction. The immunoreactivity of the antibodies was measured as the ratio of the area occupied by fluorescent cells (thresholded area) to the total area of the ventral horn. A minimum of 5 animals per condition were analyzed. Images were captured using a CoolSNAP camera (Photometrics, Tucson, USA) coupled to an ECLIPSE Ti-S microscope (Nikon, Tokyo, Japan). Images were processed using MetaMorph 7.6.5.0 analysis software (Molecular Device, San Jose, USA). For colocalization analysis between Iba-Kv1.3, we used the *Measure Colocalization* plugin of Metamorph 7.6.5.0 image analysis software (Molecular Device, San Jose USA). First, 10X magnification images of ventral horns of the lumbar spinal cord were obtained. Pixel-to-micron calibration was performed for all acquisitions using *Calibrate Distances* and inclusive threshold was defined for each channel to subtract background. The analysis was performed on active regions (ventral horns). Within the *Measure Colocalization* dialogue box, we selected the images, and the percentage of the area covered by the overlap of Kv 1.3 and Iba1 signals was calculated.

For MN survival, the entire ventral horn of the lumbar cord was imaged at × 10 magnification and the number of MNs was evaluated as previously described (Cocozza et al., 2018).

### 2.5 Serum cytokine assay

Blood samples were collected by cardiac puncture immediately before the perfusion and sera were obtained by centrifugation at 2000 × g, at 4°C for 10 min The serum cytokine analysis was performed with pooled serum samples in equal proportions for 14 mice per group, obtaining four replicates for each condition in two independent experiments according to the manufacturer’s instructions. (Proteome Profiler™ Array Mouse Cytokine Array Panel A; R&D Systems; ARY006). Samples were measured on the ChemiDocTM MP Blot reader system (BioRad; Hercules, Calif.). Image Lab Software (BioRad; Hercules, Calif.) was used for data analysis.

### 2.6 Cell cultures and oxygen consumption rate (OCR) assay

NSC-34 cells and NSC-34 cells expressing hSOD1G93A (named G93A) were kept in culture in high-glucose Dulbecco’s modified Eagle’s medium supplemented with FBS (5%, Euroclone), 1 mM glutamine, 1 mM pyruvate, antibiotics (100 IU/mL penicillin and 100 μg/mL streptomycin), and G418 sulfate (0.5 mg/mL) (Invitrogen). Real-time measurements of OCR were performed using an XFp Extracellular Flux Analyzer (Seahorse Bioscience, North Billerica, MA, USA) as already described. NSC-34 and G93A cells with or without PAP-1 (25 or 50 nM) were plated in the sensor cartridges (Seahorse Bioscience; 10,000 cells/well) and cultured in standard condition 1 week before measurements. OCR was evaluated in XF media composed of DMEM medium containing 10 mM glucose, 1 mM sodium pyruvate, and 2 mM L-glutamine under basal conditions and in response to 2.5 μM oligomycin, 1.5 μM carbonylcyanide-4-(trifluoromethoxy)-phenylhydrazone (FCCP), and 1 μM antimycin and rotenone. Indicators of mitochondrial respiratory function were calculated from the OCR profile: basal OCR (before the addition of oligomycin), ATP-bound OCR (calculated as the difference between the basal OCR rate and the OCR rate induced by oligomycin), and maximal OCR (calculated as the difference between the rate of FCCPs and the rate of antimycin + rotenone). Experiments using the Seahorse system were performed with the following assay conditions: 3 min mixing; 3 min waiting; and 3 min measurement.

### 2.7 Mitochondria analysis

For immunohistochemistry analysis WT or hSOD1G93A lumbar spinal cord sections were permeabilized for 45 min in PBS1X containing 0.3% Triton X-100 and 15% normal goat or donkey serum and then immunolabeled overnight or 3 overnight at 4°C using the following primary antibodies: rabbit-TOMM20 (Abcam, 186735,1:500) and mouse SMI-32 (BioLegend, 1:500), and the following secondary antibodies: anti-rabbit A488 (Invitrogen, 1:300) and anti-mouse A647 (Invitrogen, 1:300). Optical Sections were acquired every 0.4/0.6-μm with SP8 laser scanning confocal system (Leica) with a 63X objective (NA 1.4) as already described (Petrelli et al., 2023). Images were analyzed using Imaris (Bitplane AG, Zurich, Switzerland) software as already described (Zehnder et al., 2021). The acquired stacks were transformed to surfaces (3D reconstruction) using Imaris (9.1, Bitplane AG, Zurich, Switzerland) and the “new surface” option by selecting the region of interest around a single motorneuron and adjusting the threshold (without background subtraction) in such a way as to avoid creating surfaces over noise and ensure that everything considered real fluorescence was covered by a gray surface and plotted using Excel and GraphPad Prism 9 software (GraphPad). At least 3-5 neurons per mouse were analyzed, and the percentage of all of the individual neurons from the same mouse was pooled. The images and the 3D reconstruction were exported and adjusted using Adobe Photoshop CS5 (Adobe System Incorporated, San José, California, USA) software.

### 2.8 Statistical analysis

Data are expressed as the mean ± standard error of the mean (S.E.M.). Unpaired Student’s *t*-test, one-way or two-way analysis of variance (ANOVA) was performed. Survival analysis was performed by Kaplan-Meier survival analysis. A value of P< 0.05 was considered significant. All statistical analyses were carried out using the Sigma Plot 11.0 Software (Systat Software GmbH, Erkrath, Germany) and GrahPad version 8.0 e 9.0.

## 3 Results

### 3.1 Disease onset evaluation in B6SJL-TG (SOD1*G93A)1GUR/J mice

**FIGURE 1:**
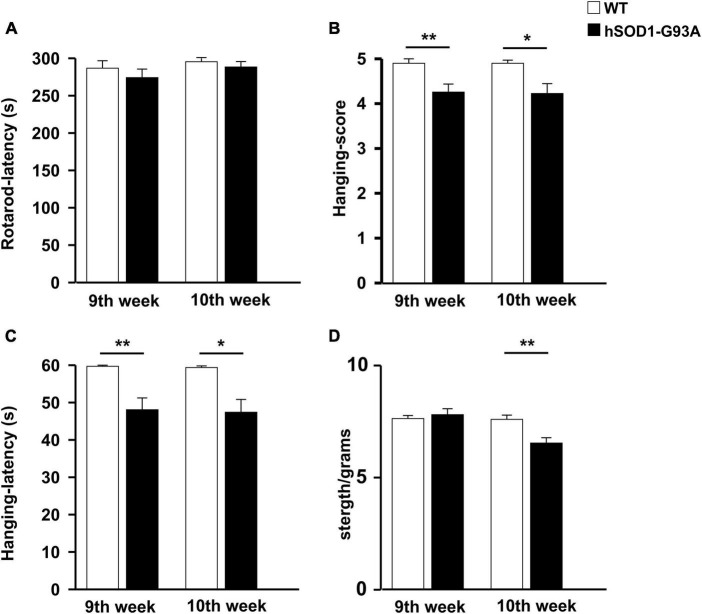
Disease onset evaluation. Histograms representing the performance of WT (*n* = 20, white) and hSOD1G93A (*n* = 31, black) animals at 9 and 10 weeks of age on the rotarod **(A)**, hanging wire test **(**score, **B)**, and latency **(C)**, strength test (normalized for animal weight) **(D)**. Data are the mean ± standard error (SE); *p < 0.05, **p < 0.01, Unpaired Student’s *t*-test. Three independent experiments.

In humans, ALS is characterized by a phenotype dependent on progressive neuronal loss and degeneration. The clinical signs of ALS are typically represented by adult-age onset of weakness in the limb muscles. Disease progression leads to muscle atrophy and impairment of motor function till respiratory failure (Masrori and Van Damme, 2020). To characterize the ALS phenotype of the mouse model used in this study, we assessed the growth rate and locomotor activity in B6SJL-TG (SOD1*G93A)1GUR/J and non-transgenic littermates starting from the 7*^th^* week of age. We have previously investigated the progression of sign appearance in a different G93A SOD1 strain, the B6.Cg-Tg(SOD1-G93A)1Gur/J line (Cocozza et al., 2018). Mice were studied longitudinally until they were not able to straighten themselves within 30 s after being placed on either side. A two-way ANOVA was used to evaluate the effects of genotype and time on mean growth rate (Supplementary Figure 1A). This analysis revealed that the growth rate of hSOD1-G93A mice was significantly lower compared to WT littermates [F(1,263) = 6.280, *p* = 0.013]. Moreover, hSOD1-G93A showed a progressive decrease in motor performance over time compared to WT mice in the rotarod test [F(1,258) = 29.830 *p* < 0.001, Supplementary Figure 1B], in the hanging wire test [F(1,272) = 23.983 *P* < 0.001; [F(1,282) = 15.047 *p* < 0.001; Supplementary Figures 1C, D], and in the grip test [F(1,271) = 28.627 *p* < 0.001; Supplementary Figure 1E]. Unpaired Student’s t-test revealed that WT and hSOD1-G93A significantly differ in the hanging wire test starting from the 9*^th^* week of age [*t* = 642.000, *p* = 0.003, Figures 1B, C] and in grip strength starting from the 10*^th^* week of age [*t* = 3.182, *p* = 0.003; Figure 1D]. No differences were detected in the rotarod test at the 9*^th^* and 10*^th^* weeks of age (Figure 1A). Based on these results, we identified the early onset of the disease between the 9*^th^* and 10*^th^* weeks and established this time window for the beginning of treatment.

### 3.2 Kv1.3 channel inhibition improves motor sign progression and survival in hSOD1G93A mice

**FIGURE 2:**
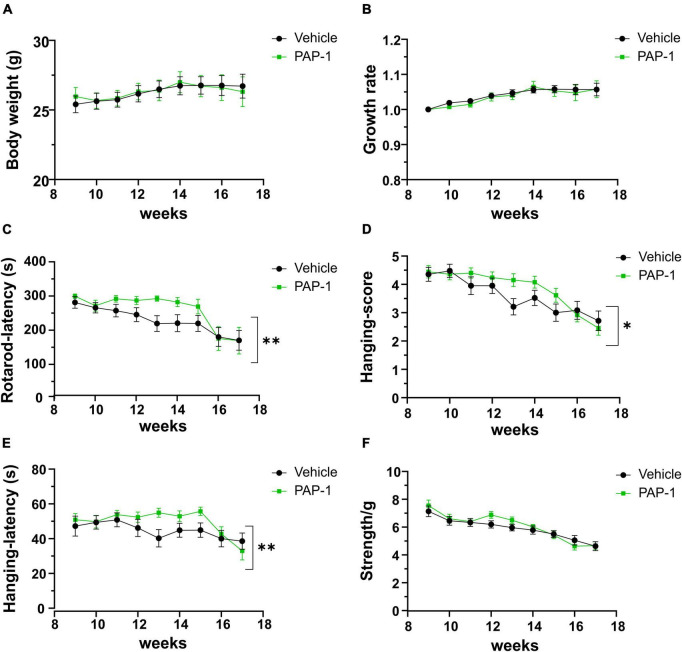
Effect of PAP-1 on motor activity: Body weight, growth rate **(A,B)** and Behavioral tests **(C–F)** of hSOD1G93A mice treated with PAP-1 (40 mg/kg) (green squares, *n* = 18) and with vehicle (black circles, *n* = 21); panel **(C)** Rotarod test, panel **(D)** Hanging Wire Test score panel **(E)** Hanging Wire test latency, panel **(F)** Grip strength test normalized with weight (g). Data are expressed as Mean ± SE; *p < 0.05, **p < 0.01, by Two-way ordinary ANOVA.

**FIGURE 3:**
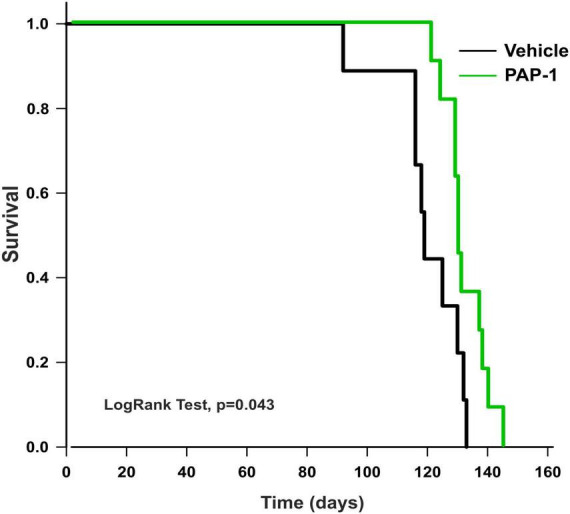
Analysis of survival (days) of hSOD1G93A mice treated with PAP-1 (40 mg/kg) (green line, *n* = 11) and vehicle (black line, *n* = 9). Kaplan-Meier, LogRank Test, *p* = 0.043.

To investigate a possible role of Kv1.3 channels on motor sign progression and survival in hSOD1G93A mice, animals were treated with the brain penetrant Kv1.3 antagonist PAP-1 (40mg/kg, i.p.) 5 days a week, starting between the 9*^th^* and the 10*^th^* week of age, when mice were early symptomatic. Mice were monitored weekly and tested in the rotarod test, hanging wire test, and grip force test, as described in the method section. Inhibition of Kv1.3 channels did not affect the weight or the growth rate of hSOD1-G93A mice compared to the vehicle group [F(1,312) = 0.021 p = 0.882, F(1,314) = 0.536 *p* = 0.464, respectively; Figures 2A, B]. However, PAP-1 administration significantly improved the performances of hSOD1-G93A mice in the rotarod test [F(1,316) = 7.935, *p* = 0.005; Figure 2C] or to hanging wire test [F(1,391) = 4.713, p = 0.030; F(1,361) = 6.801 *p* = 0.0095, Figures 2D, E] compared to the hSOD1-G93A control group. Notably, no clear improvement was observed in the grip strength test in treated mice [F(1,409) = 1.870, *p* = 0.172, Figure 2F], even though PAP-1 mice showed an increase in strength between weeks 12 and 14 (12 week vehicle vs. 12 week PAP-1, *p* = 0.0597 by Student’s *t*-test). Weekly analysis of rotarod and hanging wire test data (Figures 2C–E) showed that the beneficial effects of PAP-1 were maximal at week 13 compared to vehicle (13 weeks vehicle vs. 13 weeks PAP-1 for rotarod *p* = 0.0142, hanging wire test score and time *p* = 0.0124 and *p* = 0.0102 by Student’s *t*-test), highlighting an interesting time window for a potential next study on the role of KV.13 in ALS. Of particular interest, the administration of PAP-1 at the symptomatic stage significantly prolonged the survival time of hSOD1-G93A treated mice compared to the vehicle group (vehicle 120.11 ± 4.1; PAP-1 130.18 ± 2.1) (Figure 3). Altogether, these results suggest that the inhibition of Kv1.3 channels ameliorated the motor coordination and reduced the muscle and limb strength impairment typical of ALS disease, as well as delayed the disease progression, prolonging the survival time in hSOD1-G93A modeling ALS phenotype.

### 3.3 Kv1.3 channel blockade reduces glial cell reactivity in the spinal cord of hSOD1-G93A mice

**FIGURE 4:**
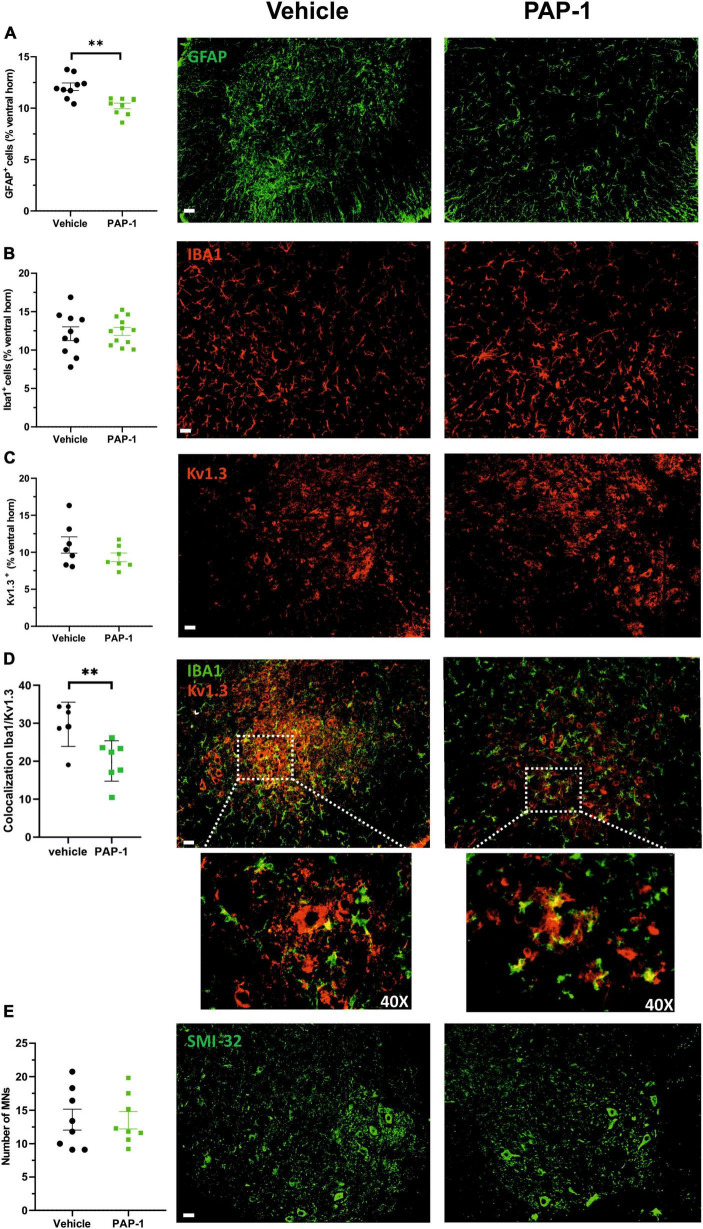
**(A)** Mean area of GFAP + cells (expressed as % of spinal cord area) in hSOD1G93A mice (17–18 weeks old) treated with vehicle (oil) or PAP-1. Each circle represents one mouse (*n* = 9 mice per condition, **p < 0.001 two-tailed Student’s *t*-test). Right: representative immunofluorescence image of GFAP+ cells in spinal cord sections. Scale bar: 50 μm. **(B)** The mean area of Iba1+ cells (expressed as % of spinal cord area) in hSOD1G93A mice (17–18 weeks old) treated with vehicle (oil) or PAP-1. Each circle represents one mouse (*n* = 10–12 per treatment). Right: representative immunofluorescence image of Iba1+ cells in the ventral horn of spinal cord sections. Scale bar: 50 μm. **(C)** Kv1.3+ immunoreactivity (expressed as % of spinal cord area) in hSOD1G93A mice (18 weeks old) treated with oil or PAP-1. Each circle represents one mouse (*n* = 7 mice per treatment). Right: representative immunofluorescence image of Kv1.3+ staining in spinal cord sections. Scale bar: 50 μm. **(D)** Colocalization of Iba1 and Kv1.3 covered area in the spinal cord in hSOD1G93A mice (17–18 weeks old) treated with vehicle (oil) or PAP-1. (*n* = 6–7, **p < 0.01 two-tailed Student’s *t*-test). Right: representative immunofluorescence image showing the microglial expression of Kv1.3 as indicated by white arrows. Scale bar: 50 μm. Dashed square: 40x magnification of co-localization signals **(E)** Quantification of Smi32+ MNs in the ventral horns of the spinal cord in hSOD1G93A mice treated with vehicle (oil) or PAP-1 (*n* = 8 per treatment). Scale bar: 50 μm. Right: representative immunofluorescence image of Smi32+ – MNs in spinal cord sections. Scale bar: 50 μm. All data are expressed as mean area ± SE.

Accumulating evidence described inflammatory events in ALS patients and animal models, including the presence of reactive astrocytes and microglia, and the infiltration of peripheral lymphocytes, natural killer (NK) cells, and macrophages, which play determinant roles in disease pathogenesis (Peric et al., 2017; Béland et al., 2020; Clarke and Patani, 2020; Garofalo et al., 2020, 2022; Vahsen et al., 2021). In this scenario, we investigated whether Kv1.3 channel activity could be relevant for disease progression in hSOD1-G93A mice. We report that the abundance of glial fibrillary acidic protein (GFAP) + cells decreased in the lumbar spinal cord of hSOD1-G93A mice upon PAP-1 treatment (Figure 4A), suggesting that Kv1.3 channels may affect the inflammatory phenotype of astrocytes (Peric et al., 2017). Moreover, the expression of the ionized Ca^2+^-binding adapter molecule 1 (Iba1, a widely used microglia/macrophage marker) and the Kv1.3 channels were analyzed in the ventral horns of the spinal cord upon PAP-1 treatment. We observed that the area occupied by Iba1 signals in the spinal cord did not change between the groups (Figure 4B). Similarly, the Kv1.3 channel staining showed no significant difference upon PAP-1 treatment of hSOD1-G93A mice (Figure 4C). However, PAP-1-treated mice had reduced Kv1.3/Iba1 co-staining, suggesting lower expression of the channel on microglial/macrophage cells (Figure 4D). The beneficial effects of PAP-1 on the motor activity of hSOD1G93A mice were not associated with a reduction of motor neuron degeneration, because no differences were observed in the number of Smi32+ cells in the lumbar spinal cord of hSOD1-G93A mice (Figure 4E). Finally, we also investigated the number of peripheral lymphocytes in the lumbar spinal cord of hSOD1-G93A mice after PAP-1 treatment at later stages of the disease. However, we did not find a statistical difference in the number of CD8-positive cells per ventral horn (vehicle 10.31 ± 0.92; PAP-1 12.62 ± 0.76, *p* = 0.061 by Student’s *t*-test). Taken together, these data suggest that Kv1.3 activity in the hSOD1G93A mice is involved in astrogliosis and microglia activation without affecting CD8-positive cell infiltration.

### 3.4 Systemic inflammation is reduced in PAP-1-treated hSOD1-G93A mice

**FIGURE 5:**
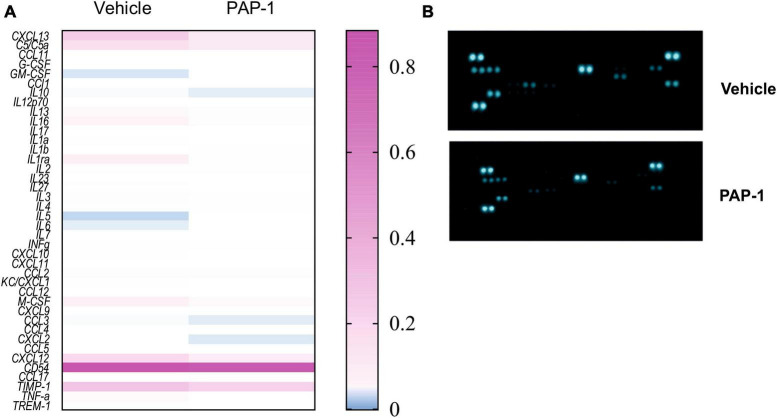
**(A)** Heatmap for the inflammatory factor levels measured in the serum of hSOD1G93A mice treated with PAP-1 or vehicle, *n* = 4 from 14 pooled samples per condition, two independent experiments; **(B)**: representative immune-array dot blots of hSOD1G93A mice sera.

It has been reported that hSOD1-G93A mice and ALS patients have increased plasma levels of inflammatory cytokines (Hu et al., 2017). We investigated the effect of PAP-1 treatment on serum cytokine levels of hSOD1-G93A mice and observed a reduction of some inflammatory factors in comparison with the vehicle-treated group, as shown in Figure 5. The specific variation for each cytokine is reported in Table 1, as fold change (the ratio between the normalized chemiluminescence intensity of PAP-1 over vehicle spots) upon Kv1.3 inhibition. Fourteen factors out of 44 were significantly reduced: among them, interleukins such as IL-1rα IL-1β, IL-3, IL-6, IL-7, and IL-16, chemokines, including CCL1, CXCL1, CXCL2, and CXCL11-13, the cytokine TNF-α and, the soluble form of CD54. These data show that inhibition of Kv1.3 channels modulates the inflammatory profile of mice, affecting important blood inflammatory factors in human ALS, including IL-1 significantly correlated to disease severity (Jin et al., 2020).

**TABLE 1:** Inflammatory cytokines modulated by KV1.3 inhibition: Fold change of levels of cytokines, chemokines, and immunomodulatory factors in the serum of PAP-1 treated hSOD1G93A mice compared to vehicle-treated mice.

Cytokine	Mean PAP-1/Vehicle ± se		*P*-Value
**TNF-α**	0.267 ± 0.012	****	<0.000001
**sICAM-1/CD54**	0.968 ± 0.005	*	0.023533
**I309/CCl1/TCA3**	0.443 ± 0.081	*	0.023532
**IL1ra**	0.159 ± 0.037	***	0.00005
**IL7**	0.434 ± 0.025	**	0.002928
**IL16**	0.420 ± 0.060	**	0.00296
**IL1β**	0.216 ± 0.109	*	0.010608
**IL6**	0.203 ± 0.118	*	0.030169
**IL3**	0.615 ± 0.075	*	0.040584
**KC/CXCL1**	0.285 ± 0.005	***	0.000014
**SDF-1/CXCL12**	0.568 ± 0.064	*	0.011748
**MIP-2/CXCL2**	0.148 ± 0.126	*	0.011948
**I-TAC/CXCL11**	0.351 ± 0.062	*	0.017334
**BLC/CXCL13/BCA1**	0.411 ± 0.129	*	0.047472

Data are expressed as PAP-1 to Vehicle ratio ± SE, *n* = 4 from 14 pooled samples per condition, two independent experiments, *p*-values obtained by Multiple *t*-test.

*p < 0.05,

**p < 0.01,

***p < 0.001,

****p < 0.0001.

### 3.5 Improving mitochondrial morphology and function in hSOD1G93A motor neurons through Kv1.3 channel inhibition

**FIGURE 6:**
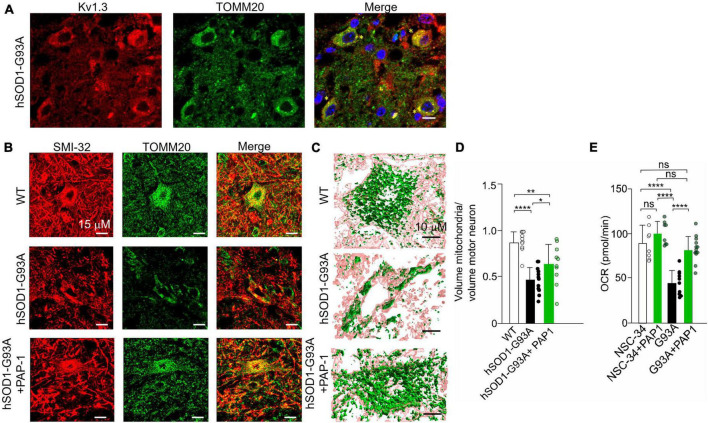
**(A)** Representative single plane confocal images of KV1.3 (red), TOMM20 (green), merge channels, and Hoechst staining (blue) for nuclei visualization in lumbar spinal cord region of hSOD1-G93A mice at early symptomatic stage (10 weeks old). 60x objective (scale:10 um) yellow stars indicate the co-localizing signals; **(B)** Representative images of the mitochondrial network in alpha motor neurons of healthy WT littermates, hSOD1G93A mice treated with vehicle or PAP-1 at late symptomatic stage (17–18 weeks old). **(C)** 3D confocal reconstruction of the mitochondrial network in single motor neurons labeled with SMI-32 (pink), and with anti-TOMM20 antibody (green). **(D)** Quantification of the cytosolic volume occupied by the mitochondrial network in WT, hSOD1G93A mice treated with vehicle or with the potassium channel inhibitor PAP-1. Data are expressed as mean ± SD *n* = 11–12 cells, 3 animals per group, by One way ANOVA. **(E)** OCR experiments: quantification of the ATP-linked OCR in NSC-34 and NSC-34 cells expressing hSOD1-G93A (G93A) treated with PAP-1 for 72 h. Values are expressed as mean ± SD of three independent experiments conducted in quadruplicate on each cell line, *n* = 12 ****p < 0.0001 by One way ANOVA. *p < 0.05, **p < 0.01.

Kv1.3 channels have been localized in the mitochondrial membrane of T lymphocytes (Gulbins et al., 2010) and cancer cells (Leanza et al., 2012). Initially, we examined the distribution of Kv1.3 in the lumbar spinal region of hSOD1G93A mice. As depicted in Figure 5A, Kv1.3 immunostaining exhibits a punctate pattern and is broadly distributed throughout the neuropil, consistent with previous observations (Otuyemi et al., 2023). A more comprehensive analysis reveals evident co-localization of Kv1.3 with a mitochondrial marker, TOMM20. These findings suggest that motor neurons likely express the channel on the mitochondria (Figure 6A). We then sought to determine whether Kv1.3 channel activity might play a role in the process of mitochondrial degeneration in hSOD1G93A motor neurons. To investigate this, we conducted confocal fluorescence analysis (Figure 6B) and 3D reconstruction following the labeling of motor neurons with the anti-neurofilament H non-phosphorylated antibody (SMI-32) and mitochondria with the TOMM20 (Figure 6C). The results, as shown in Figure 6D, indicate that motor neurons from hSOD1G93A mice treated with PAP-1 exhibit a more intact mitochondrial network compared to mice treated with vehicle alone. This suggests that Kv1.3 channel activity is implicated in the mitochondrial degeneration observed in hSOD1G93A mice. Finally, to assess the functional aspects of neuronal mitochondria following chronic inhibition of Kv1.3 channels, we conducted oxygen consumption rate (OCR) experiments using motor neuron lines NSC-34 and NSC-34-G93A carrying the mutation of hSOD1 (Tartari et al., 2009). Treatment with PAP-1 (25 nM for 3 days) improved the mitochondrial respiratory function of NSC34-G93A cells, increasing the rate of oxygen consumption to levels comparable to healthy NSC-34 cells (Figure 6E). Taken together, these findings suggest the involvement of Kv1.3 channels in ALS associated with mitochondrial dysfunction.

## 4 Discussion

Increasing evidence suggests that Kv1.3 channels could represent a target of interest for the development of novel therapeutics for neurodegenerative diseases (Rangaraju et al., 2009; Chen et al., 2017; Maezawa et al., 2018; Sarkar et al., 2020; Cojocaru et al., 2021), epilepsy (Zhang et al., 2022), and glioma (Grimaldi et al., 2018).

In the present study, we investigated the role of these voltage-dependent K^+^ channels in one ALS mouse model at the symptomatic stage of the disease, between the 9*^th^* and the 10*^th^* week of age, and described the effect of its chronic inhibition on motor activity and survival. We found that channel activity is involved in the impairment of motor coordination as well as hindlimb muscle dysfunction over time because its inhibition with PAP-1 improved both the Rotarod and hanging wire test performance compared to vehicle-treated hSOD-G93A mice. Furthermore, even though we started to administer the Kv1.3 inhibitor when the disease was already established, treated mice showed a significant increase in mean survival. Because of the advanced pathology at first symptom onset in this model, the ability to have a beneficial effect on motor function and lifespan is remarkable. In hSOD1-G93A mice, several studies targeting different pathomechanisms showed large effects on motor symptoms and survival at a pre-symptomatic stage, and smaller or no effects when treated at symptom onset (Gotkine et al., 2015; Rabinovich-Nikitin et al., 2016; Günther et al., 2017; Tungtur et al., 2021).

High levels of microglial Kv1.3 expression are associated with a pro-inflammatory profile of microglia in various neuropathological conditions, including Alzheimer’s disease, Parkinson’s disease, multiple sclerosis, and stroke, and microglial pro-inflammatory responses are significantly reduced by pharmacological blockade or genetic deletion of microglial Kv1.3 (Rangaraju et al., 2009; Chen et al., 2017; Maezawa et al., 2018; Sarkar et al., 2020). Here we show that PAP-1 treatment reduced Kv1.3 expression on microglial cells in the lumbar ventral horns, suggesting a reduced pro-inflammatory phenotype in association with reduced astrocytic overactivation. Recently, it has been demonstrated that knocking out genes for astrocyte activating factors slows disease progression in hSOD1-G93A mice (Guttenplan et al., 2020) suggesting that the attenuation of reactive astrocyte response is protective in ALS. Furthermore, we show that total Kv1.3 expression did not change between the two groups, suggesting that PAP-1 mice may also have a different cell type Kv1.3 distribution due to the presence of a more complete neuronal network of mitochondria and therefore high mitochondrial Kv1.3 expression compared to vehicle-treated mice.

A meta-analysis study including 25 case-control with 812 ALS patients and 639 control subjects, showed significant elevations of peripheral blood inflammatory cytokines such as TNF-α, TNFR1, IL1β, IL-6, IL-8, and VEGF compared to healthy individuals (Hu et al., 2017). Here we show that chronic inhibition of Kv1.3 channels reduced several pro-inflammatory factors. Blocking pro-inflammatory mediators in ALS to reduce neuroinflammation and motor neuron death is a strategy that delayed the onset of symptoms and extended the survival of mutant SOD1 mice (Neymotin et al., 2009; Stommel et al., 2009; Rabinovich-Nikitin et al., 2016); clinical use of a recombinant humanized anti-interleukin-6 receptor (IL-6R) monoclonal antibody is under investigation (Maier et al., 2015; Milligan et al., 2021). We observed a significant decrease of CXCL12 in the serum of PAP-1-treated mice compared to controls. In a mouse model of AD, it has been reported that CXCL12-CXCR4 signaling may facilitate TNF-a availability to glial TNFR1. This, in turn, triggers glutamate release that eventually leads to neuronal death (Rossi et al., 2005). CXCL12 could also be involved in the increased IL-6 level in patient serum (Ono et al., 2001; Moreau et al., 2005), for example, enhancing cytokine transcription (Tang et al., 2008). Altogether, the inhibition of Kv1.3 on immune and glial cells, the reduced levels of inflammatory cytokines and CXCL12 in the blood of hSOD1-G93A mice could promote a reduction of peripheral inflammation, which may have beneficial effects on CNS inflammation and motor symptoms.

We also showed mitochondrial expression of the Kv1.3 channel in lumbar ventral horns of hSOD1-G93A mice and demonstrated that mice treated with PAP-1 have a more complete mitochondrial network compared to mice treated with vehicle alone. Recently, an immunolocalization study performed in subcortical brain regions of mice showed that in contrast to other Kv subtypes, preferentially located on plasma membranes, signal for Kv1.3 was detected as large cytoplasmic clusters within soma, as well as smaller puncta within dendrites. In addition, colocalization of Kv1.3 and SOD2 suggested mitochondrial compartmentalization in medium spiny neurons MSN (Otuyemi et al., 2023). In lymphocytes, Bax interaction with mitochondrial Kv1.3 is a crucial step that precedes cytochrome *c* release during apoptosis (Gulbins et al., 2010). We speculate that in our model the inhibition of the channel reduced mitochondrial degeneration possibly hampering motor neuron apoptosis. However, even though we observed a significant improvement in mice’s motor activity and survival, we did not observe a reduction in motoneuron loss at the late stage of the disease. This discrepancy could be because we started to block Kv1.3 channel activity when mice were already symptomatic. It is well known that ALS pathology begins long before symptoms appear. In a human autopsy study, ∼20% axonal motor neuron loss had already occurred in anterior roots at the “pre-symptomatic” stage. Sera from people who will develop ALS, and from pre-symptomatic members of families with ALS carrying pathogenic gene mutations, showed high levels of neurofilaments, following an already active neurodegenerative process before symptoms appear (Mitsumoto et al., 2014). In addition, we demonstrated that the blockade of Kv1.3 activity rescued the respiratory deficits in NSC-34 cells expressing hSOD1-G93A protein. In melanoma cells, it has been shown that Kv1.3 channels physically interact with Complex I of the respiratory chain and that this proximity underlies the death-inducing ability of psoralenic Kv1.3 inhibitors, such as PAP-1 (Peruzzo et al., 2021). This discrepancy could be due firstly to the different levels of metabolic activation between transformed cells and neurons, and secondly to the different mechanisms that regulate the equilibrium between plasma membrane and mitochondrial Kv1.3 in cancer cells and neurons, which balances the final cellular effect (Capera et al., 2022).

In conclusion, our data establish Kv1.3 activation as an important player in the modulation of both central and peripheral inflammation and mitochondrial functions. Targeting this channel may be an effective strategy to control the neurotoxic inflammatory signals and to hamper mitochondrial degeneration in ALS. However, it would be interesting to test whether the effect of blockade of Kv1.3 in hSOD1G93A mice could also be relevant at later stages of the disease (e.g., 13 or 14 weeks of age), when very few drugs seem to have any effect on ALS mice, thus indicating a reliable treatment success in humans (Jiang et al., 2022).

## Data availability statement

The raw data supporting the conclusions of this article will be made available by the authors, without undue reservation.

## Ethics statement

The animal study was approved by the Italian Ministry of Health (Approval No. 374/2018-PR). The study was conducted in accordance with the local legislation and institutional requirements.

## Author contributions

PR: Writing – original draft, Data curation, Formal analysis, Investigation. GC: Data curation, Formal analysis, Investigation, Writing – original draft. CP: Investigation, Writing – original draft. LB: Investigation, Writing – original draft. IC: Investigation, Writing – original draft. KM: Investigation, Writing – original draft. MS: Investigation, Writing – original draft. MR: Investigation, Writing – original draft. PB: Formal analysis, Funding acquisition, Investigation, Resources, Supervision, Writing – review and editing. SF: Writing – review and editing. HW: Resources, Writing – review and editing. CL: Funding acquisition, Resources, Writing – review and editing. GD’A: Conceptualization, Funding acquisition, Project administration, Resources, Supervision, Visualization, Writing – original draft, Writing – review and editing.
