# Supplementary material for: Reduction of inflammation and mitochondrial degeneration in mutant SOD1 mice through inhibition of voltage-gated potassium channel Kv1.3
Source: Front Mol Neurosci. 2024 Jan 16;16:1333745. doi: 10.3389/fnmol.2023.1333745 (PMC10824952; doi:10.3389/fnmol.2023.1333745)
Supplement: Supplementary file 1 [file Data_Sheet_1.PDF]

Supplementary Material

Supplementary Figures

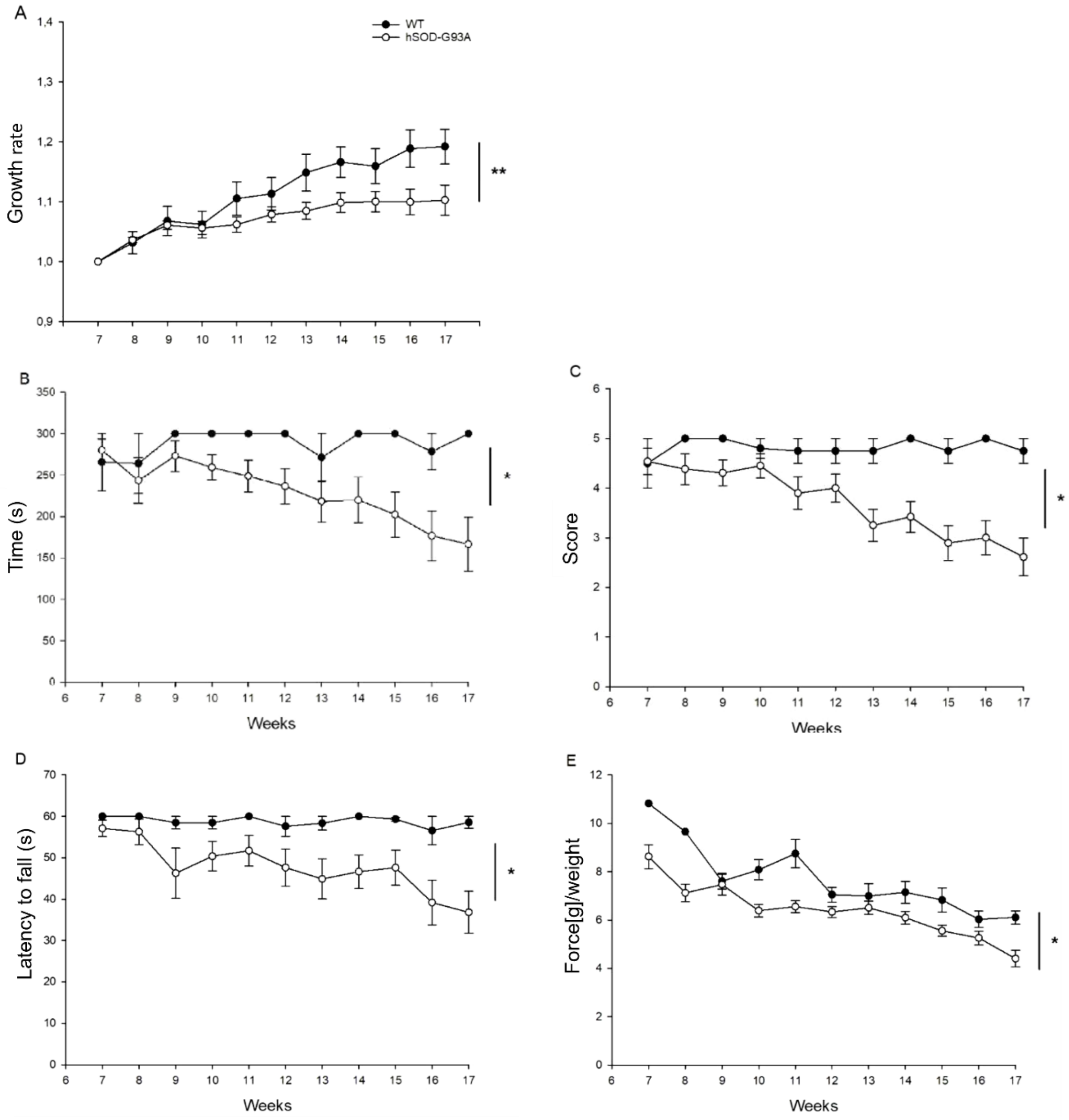

Supplementary Figure 1. A. Growth rate in WT mice (black circles, n=20) and hSOD1G93A mice (white circles, n=13). Growth rate values are normalized to body weight at 7 weeks for each mouse. B-E) Analysis of motor functions in WT and hSOD1G93A mice: rotarod test (B), hanging wire test, score (C) and latency to fall (D), grip strength test (E). Behavioral tests were performed once a week, starting at 7 weeks of age until the weeks described in the panels. Data are the mean ± standard error; *p<0.05, **p<0.01, Two-way ordinary ANOVA. Five independent experiments.
